# Modification of the Surface Crystallinity of Polyphenylene Sulfide and Polyphthalamide Treated by a Pulsed-Arc Atmospheric Pressure Plasma Jet

**DOI:** 10.3390/polym16182582

**Published:** 2024-09-12

**Authors:** Abdessadk Anagri, Sarab Ben Saïd, Cyrille Bazin, Farzaneh Arefi-Khonsari, Jerome Pulpytel

**Affiliations:** 1 LISE (UMR8235), Faculty of Science and Engineering, Sorbonne University, CNRS, 04 Place Jussieu, 75005 Paris, France; 2 COALIA, Thetford Mines, QC G6G 1N1, Canada

**Keywords:** atmospheric plasma, plasma jet, surface treatment, polymer crystallinity, heat transfer, modeling

## Abstract

Atmospheric plasma jets generated from air or nitrogen using commercial sources with relatively high energy densities are commonly used for industrial applications related to surface treatments, especially to increase the wettability of polymers or to deposit thin films. The heat fluxes to which the substrates are subjected are typically in the order of 100–300 W/cm^2^, depending on the treatment conditions. The temperature rise in the treated polymer substrates can have critical consequences, such as a change in the surface crystallinity or even the surface degradation of the materials. In this work, we report the phase transitions of two semicrystalline industrial-grade polymer resins reinforced with glass fibers, namely polyphenylene sulfide (PPS) and polyphthalamide (PPA), subjected to plasma treatments, as well as the modeling of the associated heat transfer phenomena using COMSOL Multiphysics. Depending on the treatment time, the surface of PPS becomes more amorphous, while PPA becomes more crystalline. These results show that the thermal history of the materials must be considered when implementing surface engineering by this type of plasma discharge.

## 1. Introduction

Plasma processes are renowned for efficiently modifying the extreme surface of materials to give them new properties, such as antifouling [1], anticorrosion [2], oxygen permeation barrier coating [3], or superhydrophobic [4], to name a few. These surface modifications are divided into four main processes: activation, functionalization, thin-film deposition, and etching. In the case of polymeric materials, plasma surface activation processes are commonly used before any further operation. Indeed, polymers have very low surface energies, making them unsuitable for bonding, painting, or dispersion as particles in formulations. Noeske et al. used an industrial atmospheric plasma jet system operating in open-air conditions to treat polypropylene (PP), polyethylene (PE), polyamide-6 (PA6), polyvinylidene fluoride (PVDF), and polyethylene terephthalate (PET). The surface energies increased from 27, 28, 35, 35, 35 to 52, 60, 62, 42, and 63 mJ/m^2^ for PP, PE, PA6, PVDF, and PET, respectively [5]. Numerous other studies [6,7,8] have also shown that this type of plasma source, which is easy to operate and integrate into a production line, can improve the surface energy of many polymers with very short treatment times. These increases can be explained by a rise in the surface concentration of oxygenated groups along with a change in roughness caused by surface etching, which is a competitive mechanism when processing polymers in the presence of oxygen. However, plasma is not only a source of reactive species when investigating surface chemistry. It is also a source of energy for the treated surface in the form of a flow of more or less hot gases and exothermic surface reactions such as the recombination of radicals and photons. Therefore, it has been known for a long time that plasma treatments can damage the extreme surface of the treated polymers and thus lead to the formation of the so-called weak boundary layers [9], leading to poor results for adhesion. However, one aspect that has been relatively neglected in the literature concerns the changes in surface crystallinity of plasma-treated polymers with relatively high heat fluxes. In the literature, Ben Salem et al. [6] reported some changes in the crystalline phase of PA-6 treated by a commercial plasma jet with a heat flux in the range of 100 to 300 W/cm^2^ determined by a calorimetric probe [10,11]. A modification of the crystallinity of polymers due to the rise in the surface temperature can have significant consequences because their mechanical properties depend mostly on the crystallinity level [12]. In addition, it has been reported that the semicrystalline or amorphous nature of some polymers may affect their aging behavior [13]. Therefore, plasma treatments that increase the surface temperature of polymers can improve their properties if crystallinity evolution goes in the right direction.

In this study, we report on the crystalline phase structure changes observed during the surface activation using a commercial atmospheric plasma jet system of two industrial glass fiber-reinforced polymer composites, polyphenylene sulfide (PPS, Ryton R4-220 grade, Chevron Phillips, The WoodLands, TX, USA) and polyphthalamide (PPA, Vestamid HTplus grade, Evonik, Darmstadt, Germany) containing 40% and 30% glass fibers, respectively. These composite materials are of great interest for replacing metal parts to reduce the weight of automotive vehicles. To correlate the change in the crystalline structure to the thermal history of the materials during the treatment, simulations of thermal transfers were performed using COMSOL Multiphysics software (V 5.2).

## 2. Materials and Methods

A commercial plasma torch from Plamastreat, described elsewhere [6], was used in this work. Briefly, an FG5001 generator (Plasmatreat, Les Ulis, France), with a square-wave pulsed primary voltage (17 kHz–25 kHz) from 250 to 330 V, was connected to an HTR12 transformer to ignite a pulsed arc inside the plasma torch. A swirling gas flow, with a typical flow rate of 17 to 34 SLM, was excited by passing through the arc and expelled out of the torch, forming a plasma jet. The standard PFW10 nozzle has a 4 mm diameter opening. The plasma torch was mounted on a 3-axis table, which allowed us to scan the surface of the substrate at a constant line speed and precisely control the distance between the torch nozzle and the substrate. The plasma process parameters used are listed in Table 1, and only the gas composition (air or nitrogen) and the line speed (from 5 to 30 m/min) varied in this study. 

**Figure 1:**
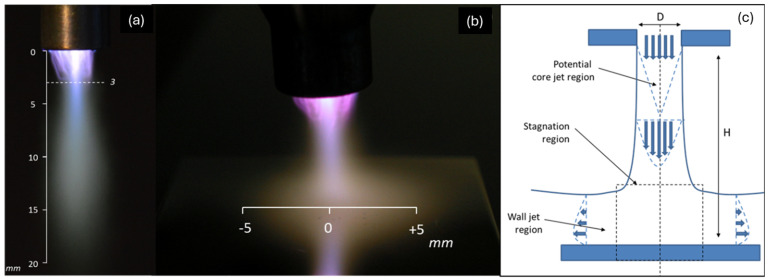
Photographs of (**a**) the free plasma jet and (**b**) the plasma jet impinging on a substrate; (**c**) flow regions of an impinging jet (Adapted with permission from [17], Elsevier, 2006).

Most atmospheric pressure plasma jet devices are homemade and therefore unique, requiring each group to carry out a complete characterization of their systems. The advantage of using a commercial system like the one in this study is the possibility to gather and cross-reference the data available in the literature to gain a complete understanding of the process. Therefore, the plasma jet shown in Figure 1 was characterized by optical emission spectroscopy [8,14], electrical measurements [7,15], or acoustic measurements [16]. The heat flux transferred by the plasma jet on the substrate and measured by a calorimetric probe [10,11] was used for comparison with our results.

## 3. Results

### 3.1. Determination of the Forced Convective Heat Transfer Coefficient

Under the processing conditions described in Table 1, the heat flux measured with the calorimetric probe [11] was 150 W/cm^2^ under the same conditions as in this study, except that the plasma cycle time (PCT) was 50%. In this work, a PCT of only 30% was used. To perform the simulation of the surface heating of the treated materials, it is preferable to determine the heat transfer coefficient (*h*) to apply Newton’s law of convective heat transfer to the substrate (Equation (1)) where *ϕ_conv_*_,0_ is the heat flux (W/m^2^).


(1) $$\phi_{conv,0}=h\cdot \left(T_{jet}-T_{surface}\right)\;[{W/m}^{2}]$$

On the other hand, by imposing a boundary condition in the form of a constant heat flux, the temperature of the substrate always increases at the same rate, while the increase in the latter slows down as the temperature approaches that of the gas. This also justifies the use of the convective heat transfer law between the plasma jet and the substrate. The convective heat transfer coefficient depends on many factors, including geometry. Thus, the thermocouple was placed in contact with a glass substrate to keep the same configuration of jet impingement on a flat surface. The measurements were compared to simulation results, which enabled the simultaneous determination of h and the gas temperature in the jet (*T_jet_*). According to the literature [10,17], it is quite justified to neglect the power received by the surface through radiation because the latter is less than 5%. Thus, the power received by the thermocouple is only in the form of convective flow, and the cooling of the thermocouple is due to both conduction and radiation. The following power balance can be written as follows:


(2) $$P_{conv}=A\cdot \phi_{conv,0}=P_{cond}+P_{rad}\;[W]$$

**Figure 2:**
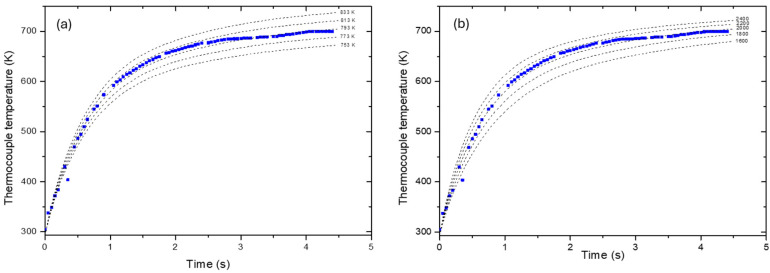
Evolution of the thermocouple temperature as a function of (**a**) *T_jet_* with *h* = 2000 W/m^2^.K and (**b**) *h* with *T_jet_* = 793 K.

In this equation, *A* is the surface area of the thermocouple immersed in the plasma jet, *P_cond_* is the power dissipated by conduction, *P_rad_* is the power dissipated by radiation, and the two fitting parameters are *h* and *T_jet_*. For the thermal properties, the K-type thermocouple was pure nickel (C_p_ = 475 J/kg.K, ρ = 7850 kg/m^3^, k = 44.5 W/m.K and ε = 0.3 are the average values in the range of 300 K–700 K). Figure 2 shows the experimentally measured temperature as well as the influence of the parameters *T_jet_* (Figure 2a) and *h* (Figure 2b) on the modeled temperature.

A good agreement was found with the experimental measurements for *h* = 2000 ± 200 W/m^2^.K and *T_jet_* = 793 ± 20 K. By applying Newton’s law with these parameters on a 1 cm^2^ surface at an initial temperature of 300 K, we obtained a heat flux of about 90 W/cm^2^, which is in good agreement with the values measured by Fröhlich and co-workers [10].

### 3.2. Modeling the Heating of a Substrate during Plasma Treatment

**Figure 3:**
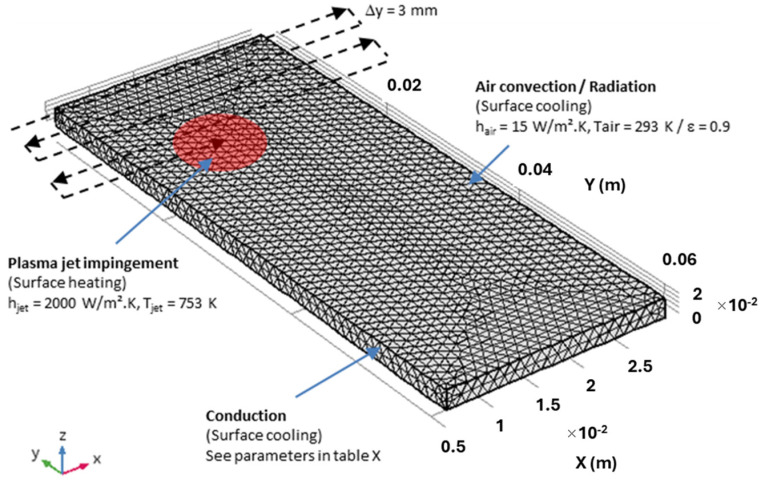
Substrate meshing, plasma torch trajectory, and heat transfer mechanisms.

COMSOL Multiphysics v5.3 was used to investigate the physics of heat transfer in solids by solving equations using the finite element method. A polymer substrate with dimensions of 65 × 25 × 2 mm is shown in Figure 3 and meshed with about 22,000 triangular elements.

The thermal properties of PPS and PPA are listed in Table 2. The size of the plasma spot determined by spatially resolved calorimetry measurements was about 10 mm in diameter [11], with a parabolic distribution. This result is in good agreement with the image analysis of the jet impact on the surface (see Figure 1).

**Figure 4:**
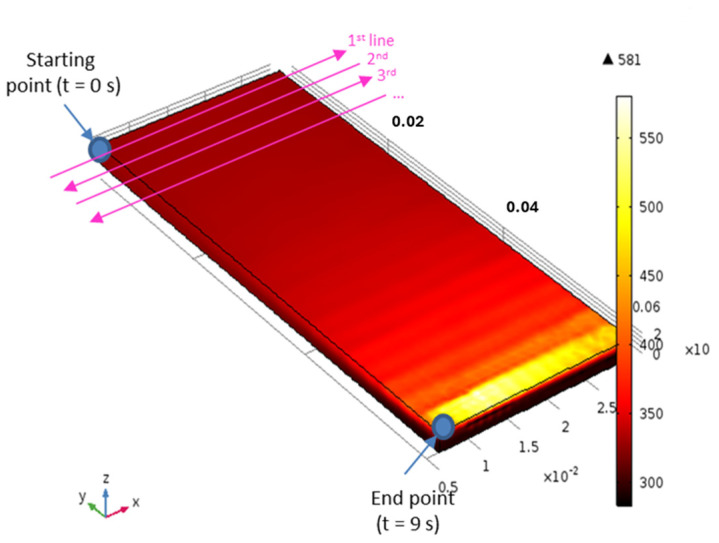
The surface temperature of PPS at *t* = 9 s. The first 4 lines of the torch pattern are also shown.

To simulate the movement of the plasma jet over the surface, a moving boundary condition was applied in the form of a convective heat flux according to the following equations:

(3) $$\phi_{conv}(x,y,t)=\phi_{conv,0}\cdot \left(1-{\left(\frac{r}{R}\right)}^{2}\right)\;[{W/m}^{2}]$$

(4) $$r=\sqrt{{\left(x-g\left(t\right).v.t\right)}^{2}+y^{2}}\;\;\;,\;r<R\;[m]$$

where *R* is the plasma spot radius, *v* is the line speed, and *g*(*t*) is a square periodic function alternately equal to +1 and −1 to simulate the movement of the torch in one direction and then to scan the surface of the substrate in the other. At the end of each line, parameter *y* is increased by a quantity Δ*y* (3 mm) to move to the next line. The plasma jet trajectory is also shown in Figure 4. As the diameter of the plasma spot was around 10 mm, each area of the sample surface was treated an average of three times as the jet scanned the surface.

The surface of the substrate cools down by heat conduction through the thickness of the material, by convection in the surrounding air, and by thermal radiation. In this simulation, the convection for the substrate cooling was considered natural, and a transfer coefficient hair of 15 W/m^2^.K was used. However, it should be noted that the turbulence induced by the jet flow at the surface accelerates the cooling of the surface, so the estimated cooling rates are probably minimum values.

## 4. Discussion

The plasma treatments of polymers, depending on the discharge chemistry, lead to surface functionalization, chain scission, cross-linking (e.g., casing), or etching, and all these mechanisms, which have been widely studied for decades, are simultaneous and competitive. To understand the surface modifications of materials by plasma treatments, it is necessary to characterize the reactive species produced in the plasma discharge. The optical emission spectroscopy of air and nitrogen plasma jets has already been carried out in the literature on identical torches operating under similar conditions. Briefly, air plasmas [5,6] are characterized by N_2_ (C^3^Π_u_-B^3^Π_g_, second positive system), N^2+^ (B^2^Σ_u_^+^-X^2^Σ_g_^+^, first negative system), NO (A^2^Σ^+^-X^2^Π, γ system), and atomic O (777.1 nm) emissions, as well as a strong NO_2_ chemiluminescence continuum (450–800 nm). Nitrogen plasmas [18] are characterized by the same emissions but with different ratios. However, the continuum chemiluminescence of NO_2_ and atomic O emission are not observed as compared to air plasma. The interaction between the plasma jet and the humidity of the ambient air can also lead to the emission of OH (A^3^Π-X^3^Σ^−^).

In parallel with the flux of reactive species, the treated samples were also subjected to a heat flux (or energy flux such as the exothermic recombination of radicals on the surface), which increased the surface temperature of the materials during treatment. It is well known that the surface temperature plays an important role in the reaction kinetics/mechanisms, but in the case of polymers, a significant rise in temperature can lead to crystalline phase transitions, which then have consequences, for instance, on the mechanical properties of the materials. Indeed, a higher degree of crystallinity in semicrystalline polymers results in harder, stiffer, and less ductile materials [19]. Simulations are therefore a powerful tool for understanding/predicting the thermal history of materials during plasma treatments, i.e., the maximum temperature reached, the heating and cooling rates, the evolution of the temperature profile in the polymer bulk, etc.

**Figure 5:**
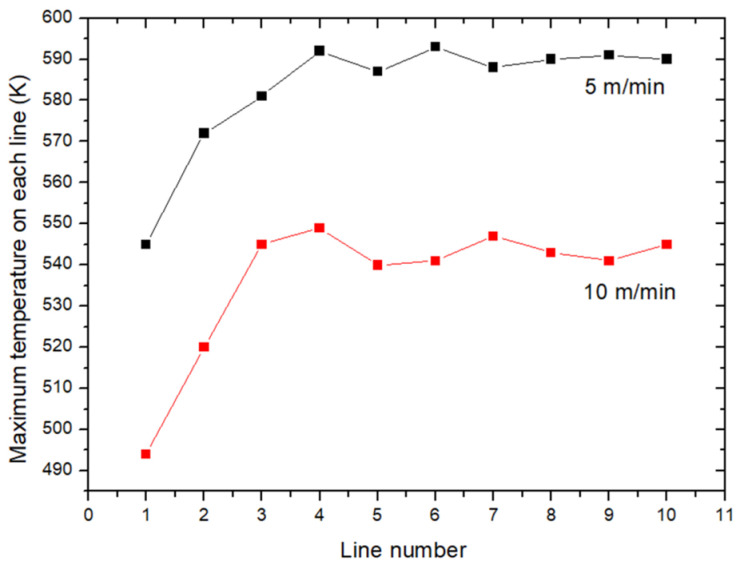
The maximum temperature on each line as a function of the line speed.

Figure 5 shows the maximum temperature reached on each line as a function of the sweep speed of the torch. Each plasma-treated line preheated the following line by the diffusion of heat into the material. However, from the third or fourth line onwards, the maximum temperature stabilized at 590 K and 540 K, respectively, for speeds of 5 m/min and 10 m/min for the rest of the treatment because the rate of the cooling of the surface by convection and radiation increased with the temperature. It is important to note that these maximum temperatures reached the melting temperatures of PPS and PPA observed by DSC around 560 K and 580 K, respectively.

**Figure 6:**
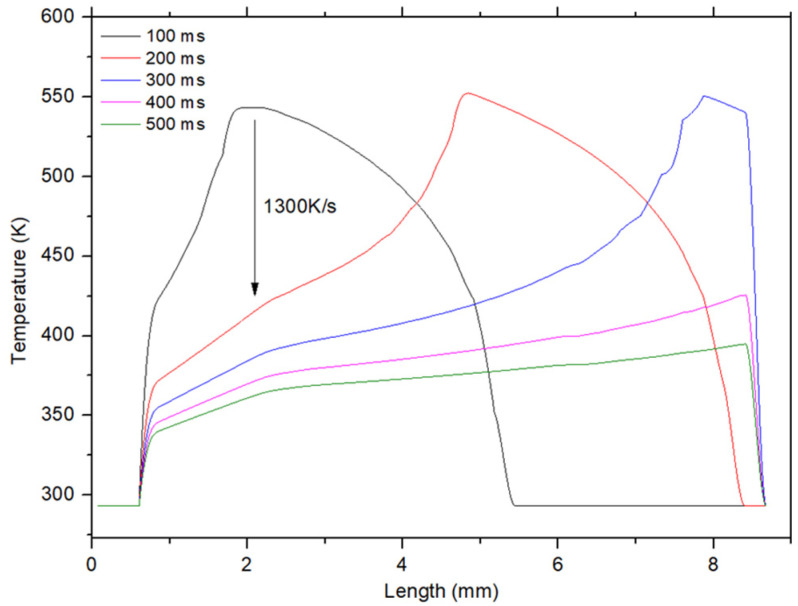
The temperature profile on a line as a function of time. The maximum temperature observed on each line corresponds to the center of the plasma jet. The line speed was 5 m/min.

The crystalline phase change in a material also depends on the cooling rate. Figure 6 shows the temperature profile on a line as a function of time and for a line speed of 5/min. At *t* = 100 ms, the surface temperature of the sample at the plasma jet was 540 K. At *t* = 200 ms, the temperature at the same point fell to 410 K, which translates into a maximum cooling rate of around 1300 K/s. The hotter the surface, the faster it cooled, so at a line speed of 10 m/min, the maximum cooling rate dropped to about 1040 K/s due to the lower temperature reached during the treatment. It can be concluded from the simulation that, under the treatment conditions used, the melting temperature of PPA and PPS can be reached locally and at the surface, and then these materials might undergo quenching due to the high cooling rate.

**Figure 7:**
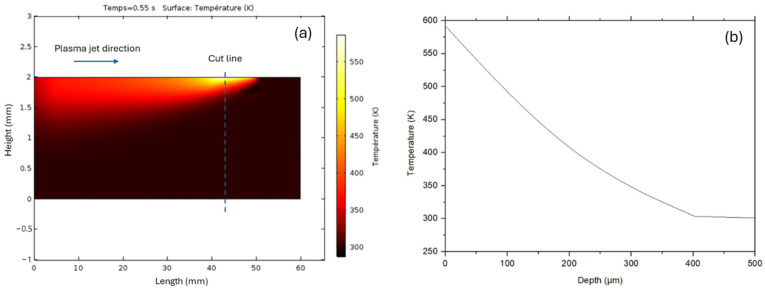
(**a**) Temperature profile along the depth of the material at a given time and (**b**) along the cut line at the same time. The line speed was 5 m/min.

It is well known that the plasma processes used to activate materials only modify the extreme surface in terms of chemical composition, but the heat can diffuse more deeply. So, the advantage of simulation is that it is possible to assess the temperature profile at depth and as a function of time, which is not possible using conventional thermal camera techniques, for example. Figure 7 shows a temperature profile according to the thickness of the PPS, and similar profiles were obtained for the PPA.

As polymers are poor conductors of heat, the latter tends to diffuse with difficulty through the material, and therefore heat is concentrated on the surface, which increases the temperature significantly. The melting temperature of PPS or PPA can be reached over a thickness of around 50 µm. It is therefore a case of surface amorphization or the crystallization of polymers. The simulation reveals that thermal conductivity is a key factor controlling surface temperature. By way of comparison, when steel (C_p_ = 910 J/kg.K, ρ = 7850 kg/m^3^, k = 44.5 W/m.K) was processed with the same conditions, the surface temperature did not exceed 60 °C (333 K) because the heat diffused rapidly through the material.

**Figure 8:**
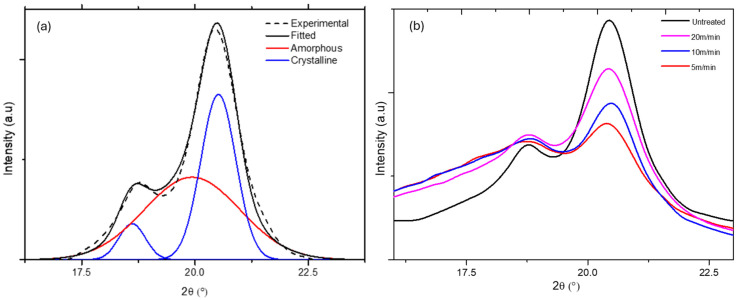
(**a**) Deconvolution of the WAXD diffractograms of untreated PPS using 3 Gaussian components at 2θ = 18.6°, 19.9° and 20.5°. The baseline was corrected by interpolation and *R*^2^ = 0.994; (**b**) diffractograms of PPS as a function of line speed.

**Figure 9:**
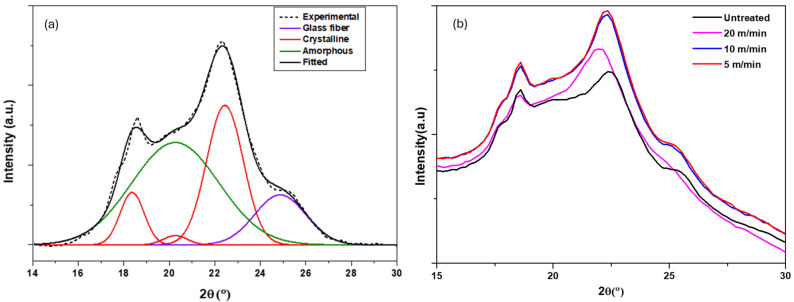
(**a**) Deconvolution of the WAXD diffractograms of untreated PPA using 5 Gaussian components at 2θ = 18.4° and 22.4°, assigned to α1 and α2 crystal, respectively, and 20.3° assigned to the γ crystal phase at 2θ = 20.2 and 24.9, assigned to the amorphous phase and glass fiber, respectively. The baseline was corrected by interpolation and *R*^2^ = 0.997; (**b**) diffractograms of PPA as a function of line speed.

To assess the effects of the thermal history (high temperature, rapid heating and cooling cycle) of plasma treatment on the polymer samples, PPS and PPA composite substrates were analyzed by wide-angle X-ray diffraction (WAXD) (Empyrean Panalytical) using the Cu Kα 1.54 Å line before and after plasma treatments. Figure 8 and Figure 9 show, respectively, the Gaussian fitting of untreated PPS and PPA diffraction patterns according to the positions of the peaks found in the literature [20,21]. The evolution of the diffractograms as a function of line speed is also shown.

**Figure 10:**
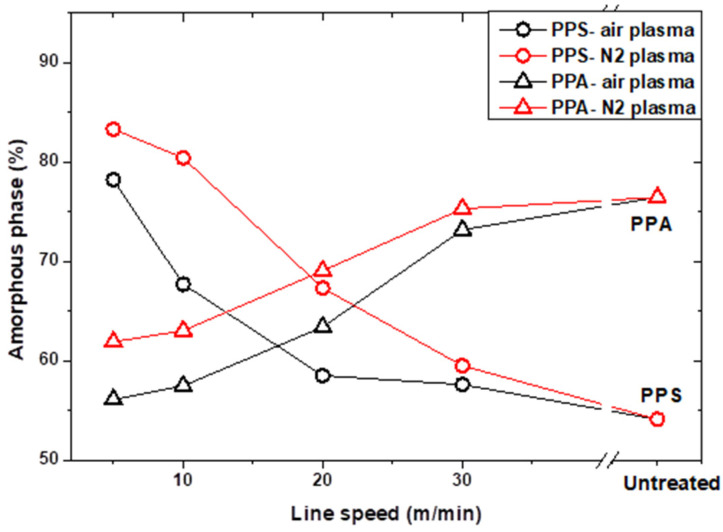
Evolution of the percentage of amorphous phase in PPS and PPA treated by air or nitrogen plasma as a function of line speed.

PPS (Ryton R4-220) and PPA (Vestamid HTplus) were initially both semicrystalline, with an amorphous phase content of 54.1% and 76.4%, respectively. The heating and cooling cycles corresponding to the scanning of the plasma torch over the substrate led to significant variations in the surface crystallinity of the polymers. As shown in Figure 10, the amorphous phase increased from 54.1% to about 85% for PPS treated with the slowest line speed (5/min), while the latter decreased from 76.4% to 56.1% in the case of PPA. The ratios of crystalline and amorphous phases are reported in Table 3 for each treatment. It can also be seen that the nitrogen plasma jets induce greater variations because the heat flux is higher than air plasma jets [11].

Table 3 also shows the maximum surface temperature reached for each treatment condition. For the same gas used (air or nitrogen), the temperature only depends on the treatment time, which is controlled by the line speed. There is a correlation between the maximum temperature reached and the phase transformation ratio of each of the polymers. It should be noted that for line speeds above 10 m/min, the simulation predicts the maximum temperatures below the melting temperature of each of the polymers. However, it is important to remember that the atmospheric plasma jet is not just a hot gas but a highly reactive medium that modifies the extreme surface of polymers. This means that materials that can melt at the surface no longer have the properties of pristine polymers. For instance, the chain scissions induced by radicals and UV can give chains greater mobility for subsequent reorganization.

The intensity of an X-ray beam penetrating a solid material decreases exponentially according to Equation (5) [22] as follows:

(5) $$I=I_{0}\cdot exp\left(-\mu .d\right)$$

where *I* and *I*_0_ are, respectively, the incident and transmitted intensity; µ is the linear absorption coefficient of the material; and d is the distance. The mean free path (λ) is simply equal to 1/µ, and it is reasonable to assume a length of 3 × λ, which corresponds to 95% of X-ray absorption. The values of µ/ρ, where ρ is the specific mass, have not been reported in the literature for PPS and PPA; however, experimental values of 6.75, 6.49, 4.25, and 3.97 cm^2^/g have been determined, respectively, for polyethylene terephthalate (PET), polymethyl methacrylate (PMMA), polystyrene (PS) and polyethylene (PE) for incident energies of 8 keV. [23]. From these data, a range of X-ray analysis depth between 200 and 300 µm can be estimated for PPS and PPA. Given the depth of surface amorphization or crystallization estimated at around 50 µm by the heat transfer model, the deeper mass of the polymer that was not altered by heat diffusion was also analyzed by XRD, which shows that the results must be analyzed with caution; that is to say, the phase transformation rate of the extreme surface is probably higher than that measured experimentally, which should be taken into account in the trend of the results.

**Figure 11:**
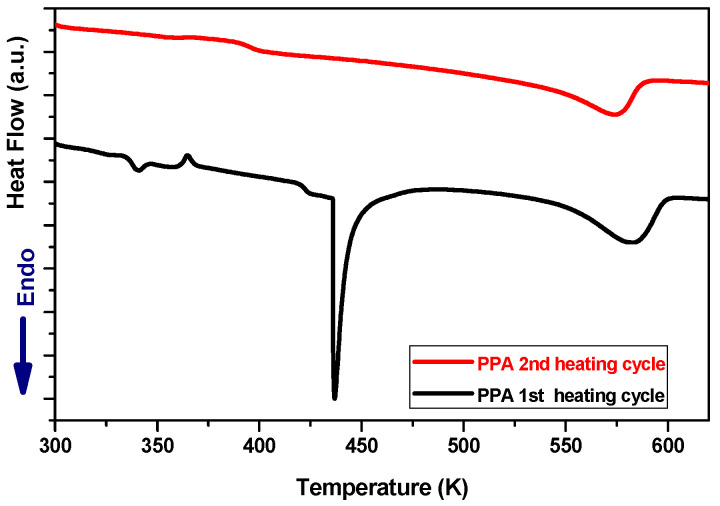
DSC curve at a heating rate of 10 K/min for PPA composite; the first and second heat scans.

**Figure 12:**
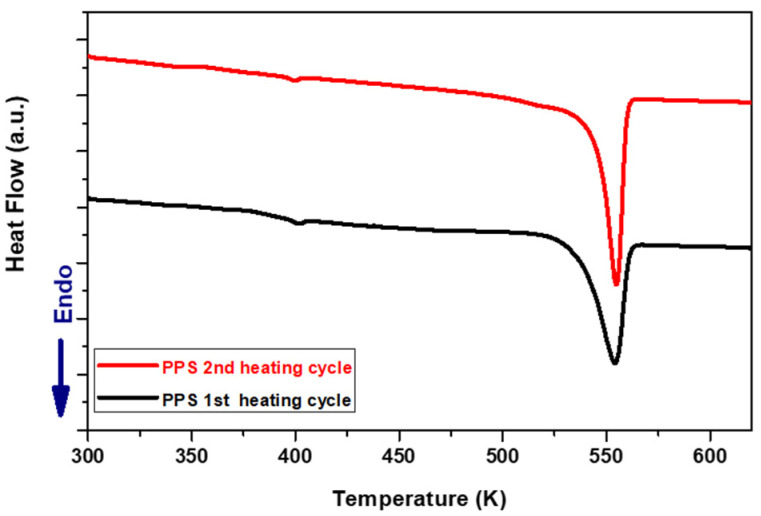
DSC curve at a heating rate of 10 K/min for PPS composite; the first and second heat scans.

To understand the plasma-induced changes in the surface crystallinity of both composites, the samples were characterized before treatment by DSC Q20 (TA instruments, Guyancourt, France). Figure 11 and Figure 12, respectively, depict the DSC diagrams of PPA and PPS before plasma treatment.

For two consecutive scan cycles, the samples were heated at a rate of 10 °C/min, from room temperature up to 350 °C and maintained for 2 min, and then cooled down to room temperature at the same rate. The estimated characteristic temperatures and crystallinity index from the second heat cycle are shown in Table 4.

Besides these characteristic temperatures, in the case of PPA, one can note the presence of a very sharp endothermic peak, which appears around 160 °C only on the first scan and disappears on the second scan (see Figure 11). By referring to the literature [20], it can be deduced that this peak could correspond to the melting point of a chain extension molecule, which is mainly added to the prepolymer to enhance its mechanical properties.

Considering the opposite tendency changes in the surface crystallinity of PPS and PPA, it is very difficult to link these changes with the thermal treatment to which the surfaces are subjected [24]. However, one can speculate that the degree of crystallinity increases for PPA because the chain extender molecule is removed by the plasma jet. Therefore, in the absence of the chain extender, the amorphous part of the polymer can undergo crystallization when treated by the plasma jet, which leads to the rapid annealing of the surface, in agreement with the results shown by modeling. Since no such molecules exist in the case of the PPS, thermal flux can cause the amorphization of PPS. This is similar to the surface amorphization observed in the case of PA6 treated with the same APPJ in air [6].

## 5. Conclusions

In summary, atmospheric pressure plasma jets can be a simple and powerful tool to modify the surface chemistry as well as the surface crystallinity of polymer composites. It is necessary to be interested in the possible modifications of the surface crystallinity of polymers because of their impact on the mechanical properties of polymers. Heat transfer modeling is a tool to estimate the possibility of a crystalline phase transformation, although the complexity of the plasma environment and the plasma–polymer interactions cannot be summarized in a simple heat transfer model. Depending on the thermal history of the composite, a relatively hot APPJ in contact with the surface may result in the significant amorphization/crystallization of the surface by continually melting or degrading the crystalline phase or by eliminating chain-extending molecules with a relatively low melting point and allowing the amorphous phase to crystallize. The main conclusions of this work are as follows:

- Thermocouple measurements coupled with simulation allowed us to obtain the parameters controlling heat transfer by forced convection between the plasma jet and the surface, i.e., *h* = 2000 ± 200 W/m^2^.K and *T_jet_* = 793 ± 20 K.
- The heat flux estimated by Newton’s law on a 1 cm^2^ surface using the aforementioned parameters is in concordance with the value reported for the same APPJ system.
- The surface temperature of the treated polymers reached their respective melting temperature (approximately 560–580 K) depending on the treatment conditions.
- Heat diffusion induced a change in the crystalline structure to a depth of approximately 50 µm under the conditions studied.
- Under the same processing conditions, polymeric materials may undergo amorphization (PPS) or conversely crystallization (PPA). However, the reason is not clear, and other molecules such as chain extenders may play a role in the formulation.
- The very rapid surface cooling rate of the order of 1300 K/s could lead to the quenching of the surface.

## Figures and Tables

**Table 1:** Process parameters.

Parameter	Value
Nozzle type	PFW10
Primary voltage	250 V
Pulse frequency	25 kHz
Plasma cycle time (PCT)	30%
Glow flow rate	1000 L/h (16.6 SLM)
Nozzle–substrate distance	10 mm
Gas type	Air or Nitrogen
Line speed	5, 10, 20 or 30 m/min

**Table 2:** Properties of PPS and PPA composites used in the COMSOL simulation.

Polymer	C_p_ (J/kg.K)	ρ (kg/m^3^)	k (W/m.K)
PPS (Ryton R4-220)	910	1700	0.31
PPA (Vestamid HTplus)	1200	1460	0.34

**Table 3:** Evolution of the percentages of the amorphous and crystalline phases of PPS and PPA as a function of the plasma gas and line speed.

Treatment	Line Speed (m/min)	Crystalline	Amorphous	Crystalline	Amorphous	Max. Surface Temp. (K) (Model)
		PPS (Ryton R4-220)		PPA (Vestamid HTplus)		
Untreated	-	45.9	54.1	23.6	76.4	-
Air	30	42.4	57.6	26.8	73.2	483
	20	41.5	58.5	36.6	63.4	500
	10	32.3	67.7	42.5	57.5	542
	5	21.8	78.2	43.9	56.1	585
Nitrogen	30	40.5	59.5	24.7	75.3	484
	20	32.7	67.3	30.9	69.1	515
	10	19.6	80.4	36.9	63.1	564
	5	16.7	83.3	38.3	61.7	612

**Table 4:** DSC calorimetric properties of PPA and PPS.

	Second Heating Cycle 10 °C/min			
Polymer	T_g_ (°C)	Tm (°C)	Δh_m_ (J/g)	χc (%)
PPA	122	270	29	21.4
PPS	87	272	23	51.5

## Data Availability

The original contributions presented in the study are included in the article/Supplementary Material; further inquiries can be directed to the corresponding author.

## Supplementary Materials

The following supporting information can be downloaded at: https://www.mdpi.com/article/10.3390/polym16182582/s1, Figure S1: Heat transfer modeling of the plasma treatment of a PPA substrate at a speed of 5 m/min under the conditions listed in Table 1.
