# Supplementary figures and images for: Modification of the Surface Crystallinity of Polyphenylene Sulfide and Polyphthalamide Treated by a Pulsed-Arc Atmospheric Pressure Plasma Jet
Source: Polymers (Basel). 2024 Sep 12;16(18):2582. doi: 10.3390/polym16182582 (PMC11435475; doi:10.3390/polym16182582)

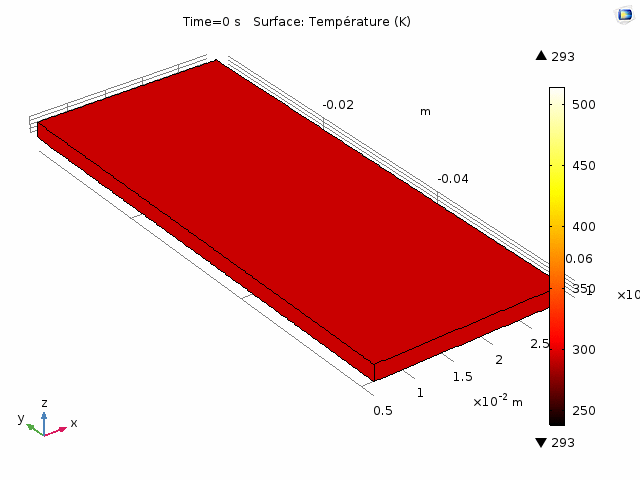

Supplement: Supplementary file 1 [file polymers-16-02582-s001.zip › polymers-3149504-supplementary.gif]
